# Population Size and Cultural Evolution in Nonindustrial Food-Producing Societies

**DOI:** 10.1371/journal.pone.0072628

**Published:** 2013-09-12

**Authors:** Mark Collard, April Ruttle, Briggs Buchanan, Michael J. O’Brien

**Affiliations:** 1 Human Evolutionary Studies Program and Department of Archaeology, Simon Fraser University, Burnaby, British Columbia, Canada; 2 Department of Anthropology, University of Missouri, Columbia, Missouri, United States of America; Universidad Autonoma de Barcelona and University of York, Spain

## Abstract

Modeling work suggests that population size affects cultural evolution such that larger populations can be expected to have richer and more complex cultural repertoires than smaller populations. Empirical tests of this hypothesis, however, have yielded conflicting results. Here, we report a study in which we investigated whether the subsistence toolkits of small-scale food-producers are influenced by population size in the manner the hypothesis predicts. We applied simple linear and standard multiple regression analysis to data from 40 nonindustrial farming and pastoralist groups to test the hypothesis. Results were consistent with predictions of the hypothesis: both the richness and the complexity of the toolkits of the food-producers were positively and significantly influenced by population size in the simple linear regression analyses. The multiple regression analyses demonstrated that these relationships are independent of the effects of risk of resource failure, which is the other main factor that has been found to influence toolkit richness and complexity in nonindustrial groups. Thus, our study strongly suggests that population size influences cultural evolution in nonindustrial food-producing populations.

## Introduction

It has long been recognized that culture is central to the adaptive success of humans [e.g., 1,2], yet only in the last few decades have substantive efforts been made to develop an explicitly Darwinian approach to the study of culture [e.g., 3–17]. Not surprisingly, therefore, a number of important topics are poorly understood, one of which is the impact of population size on cultural evolution.

Theoretical reasons exist for believing that population size affects cultural evolution. Shennan [18], for example, has shown with the aid of a population genetics model that larger populations have an advantage over smaller ones when it comes to cultural innovation because of the decreasing role of sampling effects as populations get larger. When population size is large, there is a greater probability of fitness-enhancing innovations being maintained and deleterious ones being lost than when population size is small. Similarly, Henrich [19] has demonstrated that population size can affect the probability of more complex skills being invented and maintained. In his model, learners preferentially copy the most skilled practitioner with some amount of error. The probability distribution that determines the amount of error is such that a learner will only occasionally arrive at behavior that gives a better result than the previous best. The likelihood of this occurring is partly dependent on population size because in large populations even improbable events occur occasionally and the larger the population, the more likely this is. Other authors who have reported modeling work that suggests population size can affect cultural evolution include Powell et al. [20], Premo and Kuhn [21], Mesoudi [22], and Kobayashi and Aoki [23].

The situation with regard to empirical support for the hypothesis that population size affects cultural evolution is more complicated. The reason for this is that the studies in which the hypothesis has been tested most rigorously returned conflicting results [24,25]. Kline and Boyd [24] examined the impact of population size on the marine foraging toolkits of 10 fisher–farmer groups from Oceania, and found that population size had a significant impact on both the number of tools and the average number of parts per tool. But analyses reported by Collard et al. [25] did not support the population size hypothesis. These authors tested the population size hypothesis as part of a study designed to shed light on the factors that drive variation in toolkit structure among hunter-gatherers. Collard et al.’s dataset employed comprised counts of the number of tools and tool parts in the subsistence toolkits of a worldwide sample of 20 ethnographically documented hunter-gatherer groups, plus values for a range of predictor variables, including population size. Collard et al.’s results did not support the population size hypothesis. The only variables that had a significant impact on the toolkit-structure measures were the authors’ proxies for risk of resource failure, effective temperature, and net aboveground productivity. Read [26] reanalyzed the dataset used by Collard et al. [25] and reached the same conclusion regarding the population size hypothesis.

Currently, it is impossible to identify the cause of the discrepancy between Kline and Boyd’s [24] findings and those of Collard et al. [25]. Kline and Boyd argue that Collard et al.’s results are misleading because their population estimates do not take into account intergroup contact. But there are other possible explanations for the discrepancy. For example, it might be a result of the difference in the size of the samples used in the studies. Given that significant results are more likely to be spurious with small samples than with large samples, and that Kline and Boyd’s sample is half the size of Collard et al.’s, there is a chance that it is actually Kline and Boyd’s results that are misleading. The geographic distribution of the samples is another potential explanation for the discrepancy. Kline and Boyd’s sample comprised only groups from Oceania, whereas the sample used by Collard et al. includes groups from several regions, including North America, Africa, and Australia. Thus, it is possible that the discrepancy between Kline and Boyd’s [24] findings and those of Collard et al. [25] is the result of Kline and Boyd’s [24] sample being geographically biased.

In view of the foregoing, we carried out another test of the population size hypothesis. Our study was similar to Kline and Boyd’s [24] in that we focused on the subsistence technology of food-producing groups and used Oswalt’s [27,28] method to quantify toolkit structure (see below). However, our sample was considerably larger and more geographically diverse than Kline and Boyd’s [24]. Additionally, we included data on all categories of food-getting technology, not just marine-foraging technology.

## Materials and Methods

Our sample comprised 40 nonindustrial food-producing groups from Africa, Asia, North America, South America, and Oceania (Table 1). The groups produced food primarily for subsistence rather than commercial sale and used craft-made rather than factory-produced tools.

**Table 1:** Groups in sample.

**Group**	**Country**	**Group**	**Country**	**Group**	**Country**
Akamba	Kenya	Lur	Iran	Somali	Somalia
Aymara	Peru	Malekula	Vanuatu	Tanala	Madagascar
Azande	Sudan	Mapuche	Chile	Tarahumara	Mexico
Garo	India	Mataco	Bolivia	Tikopia	Solomon Islands
Gikuyu	Kenya	Monguor	China	Trukese	Micronesia
Guarani	Paraguay	Okinawa	Japan	Tuareg	Algeria
Gwembe Valley, Tonga	Zambia	Ovimbundu	Angola	Vietnamese	Vietnam
Haddad	Chad	Pawnee	USA	Walapai	USA
Hopi	USA	Pima	USA	Yanomami	Venezuela
Jivaro	Ecuador	Pukapuka	Cook Islands	Yuma	USA
Kapauku	Indonesia	Quichua	Ecuador	Zapotec	Mexico
Kogi	Colombia	Rwanda	Rwanda	Zuni	USA
Korea	South Korea	Seminole	USA		
Lepcha	India	Sinhalese	Sri Lanka		

Present-day country names are provided as a guide to the location of the groups.

We began by calculating two measures of the toolkit structure that were developed by Oswalt [27,28]: the total number of subsistants (STS) and the total number of technounits (TTS) for each group. A subsistent is a tool that is employed directly in the acquisition of food, whereas a technounit is an “integrated, physically distinct, and unique structural configuration that contributes to the form of a finished artifact” (see p. 38 [28]). In the past, STS has been argued to be a measure of toolkit “diversity” [25,26,29], but the term “diversity” is potentially confusing. In ecology, “diversity” has two dimensions: “richness” and “evenness.” The former refers to the number of taxa in a community, landscape, or region; the latter refers to how close the taxa in a community, landscape, or region are in terms of numbers of individuals [30]. The dimension of ecological diversity that the variable “total number of subsistants present in a toolkit” is akin to is clearly “taxonomic richness.” Thus, to reduce the potential for confusion we refer here to the total number of subsistants as “toolkit richness” rather than “toolkit diversity.” There is no such ambiguity about TTS, which is generally agreed to be a measure of toolkit complexity [25–29].

When we calculated STS and TTS values, we took into account all foraging and food production-related tools used by the groups. These include tools employed in irrigation, tools used to ward off birds and mammals from agricultural fields, tools used to process food for consumption, and tools used to prepare food for storage. The main source of toolkit data was the digital version of the Human Relations Area Files (eHRAF), which is a Web-accessible, key-word-searchable collection of ethnographies [31]. Additional data were obtained from searches of hard copy ethnographic sources not included in eHRAF.

Next, we collected population size estimates for the groups, using estimates that were as close to the time period of the toolkit data as possible. In most cases, the population estimate was taken from the “Demographics” section of eHRAF’s “Culture Summary” for the group. In some cases, where the ethnographic source literature was focused on a subset of a larger cultural group (e.g., if a particular cultural group was spread across national boundaries), population size was estimated for the part of the group living in the region covered by the ethnographic source(s) from which the toolkit data were extracted. The eHRAF population estimates for a few groups were inappropriate (e.g., they were for the modern period whereas the STS and TTS values were for the late nineteenth century). For these groups, estimates were obtained from searches of relevant sources not included in eHRAF.

Subsequently, we generated values for two proxies for risk of resource failure because one of the previous tests of the population size hypothesis found that size did not affect toolkit structure when proxies of risk of resource failure were included [25]. The proxies for risk of resource failure were mean annual rainfall and effective temperature. Also known as “warmth,” effective temperature was developed to better understand the impact of temperature on the distribution of living and fossil plants [32]. It is defined as the temperature characteristic of the start and finish of the period in which plant growth occurs [32]. The effective temperature of a given location is calculated with the following equation: Effective temperature = ((18WM) - (10CM))/(WM-CM + 8), where WM is the mean temperature (in degrees Celsius) of the warmest month of the year and CM is the mean temperature of the coldest month of the year. The first constant in the equation (18) is the mean minimum temperature that will sustain tropical plant life. The second (10) is the temperature limit of polar climates for the warmest month of the year (the minimum mean temperature at the boundary between polar and boreal environments). The third (8) is the minimum mean temperature at the beginning and end of the growing season. Values for mean annual rainfall and the temperatures incorporated into effective temperature were obtained from several open-access sources of climatic information [33–37]. As far as possible, we used values for mean annual rainfall, mean temperature of the warmest month, and mean temperature of the coldest month from the same period as the toolkit and demographic data.

After compiling the dataset, we logarithmically transformed population size because the population size hypothesis predicts a concave relationship between population size and toolkit richness and complexity [18,19]. We also logarithmically transformed STS and TTS to make them more closely approximate a normal distribution. We did not need to transform mean annual rainfall or effective temperature as both variables approximated a normal distribution according to the Kolmogorov-Smirnov test (mean annual rainfall: *z* = .961, *p* = .314; effective temperature: *z* = 1.312, *p* = .064).

We then carried out two linear regression analyses. In the first we used STS as the dependent variable and population size as the independent variable. In the second we used TTS as the dependent variable and population size as the independent variable. Because multiple tests were conducted, Benjamini and Yekutieli’s [38] method of significance-level correction was used to reduce type-I error rates. We employed this method rather than the better-known Bonferroni correction because it has been shown to balance the reduction of type-I and type-II error rates better than Bonferroni correction [39].

Lastly, we conducted two standard multiple regression analyses designed to determine whether population size has an impact on the two technological variables independent of risk of resource failure. In the first we used STS as the dependent variable and population size, mean annual rainfall, and effective temperature as the independent variables. In the second we used TTS as the dependent variable and population size, mean annual rainfall, and effective temperature as the independent variables.

All analyses were carried out in PASW (SPSS) 19.

## Results

**Figure 1:**
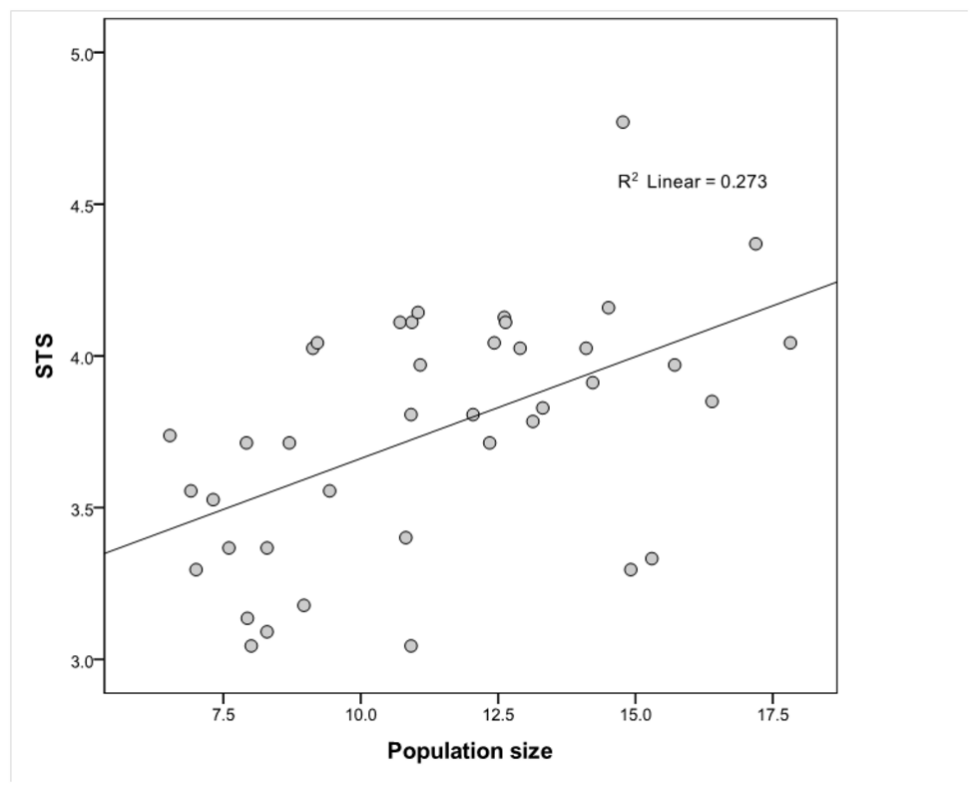
Scatter plot showing that total number of subsistants (STS) is influenced by population size in a sample of 40 small-scale food-producing groups. Both STS and population size are logged.

**Figure 2:**
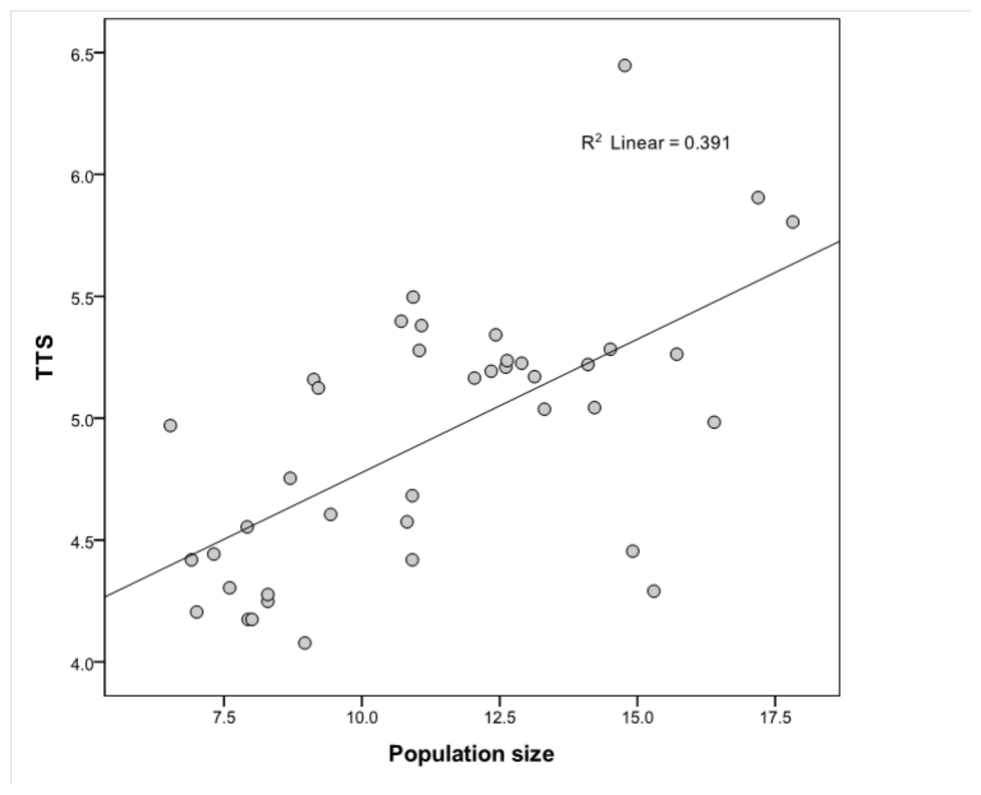
Scatter plot showing that total number of technounits (TTS) is influenced by population size in a sample of 40 small-scale food-producing groups. Both TTS and population size are logged.

A significant, positive linear relationship was obtained when STS was regressed on population size (Figure 1; *r* ^2^ = 0.27; β = 0.52; *p*= 0.001). A significant, positive linear relationship was also obtained when TTS was regressed on population size (Figure 2; *r* ^2^ = 0.39; β = 0.63; *p* < 0.000). The impact of population size on the technological variables appears to be independent of risk of resource failure. In the multiple regression analyses with STS as the dependent variable, population size was the only significant variable in the model (Table 2). Similarly, in the multiple regression analyses with TTS as the dependent variable, population size was the only significant variable in the model (Table 3). Thus, the analyses suggest that population size impacts toolkit richness and complexity among small-scale food-producing populations in the manner predicted by the population size hypothesis.

**Table 2:** Summary of results of standard multiple regression analysis carried out to assess the relative importance of population size (POP), mean annual rainfall (RAIN), and effective temperature (ET) as drivers of toolkit richness (STS) in a worldwide sample of nonindustrial food-producing societies (n = 40).

**Full model**	**POP**	**RAIN**	**ET**
*F*5.082	Beta .536	Beta .024	Beta .147
df 3, 36	t3.760	t.157	t.966
*p* .005*	*p* .001^†^	*p* .876	*p* .341
r^2^ .297	VIF 1.040	VIF 1.192	VIF 1.191

* Significant correlation using Benjamini and Yekutieli’s (2001) alpha correction (the critical value for two tests is α = 0.033).

† Significant at *p* ≤0.05.

**Table 3:** Summary of results of standard multiple regression analysis carried out to assess the relative importance of population size (POP), mean annual rainfall (RAIN), and effective temperature (ET) as drivers of toolkit complexity (TTS) in a worldwide sample of nonindustrial food-producing societies (n = 40).

**Full model**	**POP**	**RAIN**	**ET**
*F*8.396	Beta .633	Beta .047	Beta .119
df 3, 36	t4.856	t4.856	t.856
*p* .000*	*p* .000^†^	*p* .736	*p* .220
r^2^ .412	VIF 1.040	VIF 1.192	VIF 4.183

* Significant correlation using Benjamini and Yekutieli’s (2001) alpha correction (the critical value for two tests is α = 0.033).

† Significant at *p* ≤0.05.

## Discussion

The analyses reported here indicate that both the total number of subsistence tools and the total number of subsistence tool parts are positively and significantly correlated with population size in our sample of small-scale agriculturists and pastoralists. They also indicate that these relationships are independent of the effects of risk of resource. Thus, our study strongly supports the predictions of the hypothesis that population size influences cultural evolution [18–23].

The nature of our sample is such that we can evaluate the potential explanations for the fact that one of the previous tests of the population size hypothesis supports it [24] whereas the other does not [25]. To reiterate, Kline and Boyd [24] argue that Collard et al.’s [25] results are misleading because their population size estimates do not take into account inter-group contact. Another possibility is that Kline and Boyd’s [24] results are misleading because their sample is half the size of Collard et al.’s [25]. A third possibility concerns the geographic distribution of the samples used by Kline and Boyd [24] and Collard et al. [25]. Kline and Boyd’s [24] sample comprised only groups from Oceania, whereas the sample used by Collard et al. [25] includes groups from several regions, including North America, Africa, and Australia. Thus, it is possible that the discrepancy between Kline and Boyd’s [24] findings and those of Collard et al. [25] is the result of Kline and Boyd’s [24] sample being geographically biased.

The first of these potential explanations for the discrepancy between Kline and Boyd’s [24] results and those of Collard et al. [25] can be discounted in light of the present study. The population size data we discuss here are similar to the data employed by Collard et al. [25] in their test of the population size hypothesis—raw estimates provided by the groups’ ethnographers. This suggests that intergroup contact is unlikely to explain the discrepancy between Kline and Boyd’s [24] results and those of Collard et al. [25]. The sample size argument is also difficult to sustain. The sample used here includes 40 farming and pastoralist groups—a sample larger than the sample of hunter-gatherer groups used by Collard et al. [25]. Accordingly, the fact that Kline and Boyd’s [24] sample is half the size of the sample used by Collard et al. [25] cannot be responsible for the discrepancy. The idea that the discrepancy between Kline and Boyd’s [24] results and those of Collard et al. [25] is a result of Kline and Boyd’s sample being geographically biased does not hold water either, given that the sample used here is more geographically diverse than that used by Collard et al.

It appears, then, that Kline and Boyd’s [24] and Collard et al.’s [25] findings concerning the population size hypothesis are valid. The corollary of this is that the discrepancy between Kline and Boyd’s [24] results and those of Collard et al. [25] is in need of explanation. Adding the results of the present study into the mix, how is it possible for two studies to support the population size hypothesis and one study to refute it, if all the tests of the population size hypotheses reported in the studies in question are valid?

So far, we have been able to identify three potential answers to this question. The first concerns mode of production. Both samples that have supported the hypothesis comprise groups that were heavily dependent on domesticated species, whereas the sample that has refuted the hypothesis consists of groups that relied primarily on wild resources. Consequently, it could be that mode of production mediates the impact of population size on cultural evolution such that the toolkits of food-producers are more affected by population size than by risk of resource failure, whereas the toolkits of hunter–gatherers are more affected by risk of resource failure than they are by population size.

The second possibility is that hunter–gatherers possess means of reducing the negative effects of population size on cultural evolution that small-scale farmers and pastoralists do not. Recently, Henrich [40] has highlighted the potential impact of norms and institutions that foster sharing of information on the spread of inventions. It could be that information-sharing norms and institutions are stronger within small populations than in larger ones. If this is the case, then it is possible that hunter–gatherers and small-scale food-producers differ in their support of the population size hypothesis because hunter–gatherers tend to have more and/or stronger information-sharing norms and institutions than do small-scale food-producers.

The third potential answer is that there is a threshold effect in the influence of population size on toolkit structure. The hunter–gatherer groups in the sample used by Collard et al. [25] and the food-producing groups in the samples used by Kline and Boyd [24] and by us in the present study overlap in terms of size, but many of the food-producing groups are much larger than the largest of the hunter–gatherer groups (~12,000 people). Thus, it could be that population size does not have a significant impact on cultural evolution until it is greater than a value close to, or above, the upper end of the population size range for hunter–gatherers. Determining which, if any, of these hypotheses is correct will require further research of the kind reported here as well as ethnographic research and simulation studies.

In conclusion, results reported here are consistent with the hypothesis that population size has a significant impact on cultural evolution [18–23]. However, by removing doubt about results of previous studies [24,25], they also suggest that the impact of population size on cultural evolution is not as uniform as the population size hypothesis avers. It appears that population size affects cultural richness and complexity in some populations but not in others. Attempting to ascertain why this is the case is the obvious next step.
